# Abnormal Long Non-Coding RNAs Expression Patterns Have the Potential Ability for Predicting Survival and Treatment Response in Breast Cancer

**DOI:** 10.3390/genes12070996

**Published:** 2021-06-29

**Authors:** Ana Carolina Pavanelli, Flavia Rotea Mangone, Luciana R. C. Barros, Juliana Machado-Rugolo, Vera L. Capelozzi, Maria A. Nagai

**Affiliations:** 1 Discipline of Oncology, Department of Radiology and Oncology, Faculty of Medicine, University of São Paulo, São Paulo 01246-903, Brazil; ana.pavanelli@hc.fm.usp.br (A.C.P.); flavia.mangone@hc.fm.usp.br (F.R.M.); lucianalpt@gmail.com (L.R.C.B.); 2 Center for Translational Research in Oncology, Cancer Institute of São Paulo, São Paulo 01246-903, Brazil; 3 Department of Pathology, University of São Paulo Medical School (USP), São Paulo 01246-903, Brazil; jr.machado@unesp.br (J.M.-R.); vera.capelozzi@fm.usp.br (V.L.C.); 4 Health Technology Assessment Center (NATS), Clinical Hospital (HCFMB), Medical School of São Paulo State University (UNESP), Botucatu, São Paulo 01246-903, Brazil

**Keywords:** breast cancer, lncRNAs, prognosis, docetaxel, biomarkers

## Abstract

Abnormal long non-coding RNAs (lncRNAs) expression has been documented to have oncogene or tumor suppressor functions in the development and progression of cancer, emerging as promising independent biomarkers for molecular cancer stratification and patients’ prognosis. Examining the relationship between lncRNAs and the survival rates in malignancies creates new scenarios for precision medicine and targeted therapy. Breast cancer (BRCA) is a heterogeneous malignancy. Despite advances in its molecular classification, there are still gaps to explain in its multifaceted presentations and a substantial lack of biomarkers that can better predict patients’ prognosis in response to different therapeutic strategies. Here, we performed a re-analysis of gene expression data generated using cDNA microarrays in a previous study of our group, aiming to identify differentially expressed lncRNAs (DELncRNAs) with a potential predictive value for response to treatment with taxanes in breast cancer patients. Results revealed 157 DELncRNAs (90 up- and 67 down-regulated). We validated these new biomarkers as having prognostic and predictive value for breast cancer using in silico analysis in public databases. Data from TCGA showed that compared to normal tissue, MIAT was up-regulated, while KCNQ1OT1, LOC100270804, and FLJ10038 were down-regulated in breast tumor tissues. KCNQ1OT1, LOC100270804, and FLJ10038 median levels were found to be significantly higher in the luminal subtype. The ROC plotter platform results showed that reduced expression of these three DElncRNAs was associated with breast cancer patients who did not respond to taxane treatment. Kaplan–Meier survival analysis revealed that a lower expression of the selected lncRNAs was significantly associated with worse relapse-free survival (RFS) in breast cancer patients. Further validation of the expression of these DELncRNAs might be helpful to better tailor breast cancer prognosis and treatment.

## 1. Introduction

Breast cancer (BRCA) is the most common malignant tumor in females and the second leading cause of cancer-related death [1]. Although the treatment of BRCA has made great progress in recent years, the prognosis remains poor, with a 5-year survival rate of less than 60% in developing countries [2]. From a molecular perspective, breast cancer is a heterogeneous disease composed of different subtypes associated with distinct clinical prognoses. Studies in the early 2000s identified the different molecular subtypes of breast cancer that are still used today in the clinic to define prognosis and therapies: luminal A, luminal B, HER2+, and basal-like [3,4]. Despite advances in breast cancer molecular classification, there are still gaps to explain in the multifaceted presentations of breast cancer and a substantial lack of biomarkers that can better predict patients’ prognosis and response to different therapeutic strategies.

Approximately 98% of the total cellular RNA content of human cells consists of non-coding RNAs (ncRNAs) that can be classified into circular RNA (circRNA), small nuclear RNA (snRNA), nucleolar RNA (snoRNA), PIWI interacting RNAs (piRNAs), miRNA and lncRNA [5,6]. Long non-coding RNAs (lncRNAs) are defined as RNA transcripts with more than 200 nucleotides which have no protein coding ability and are implicated as an important epigenetic control of gene expression in a variety of biological processes [7]. Abnormal lncRNA expression patterns have also been reported in many malignancies, supporting their potential as novel biomarkers for molecular cancer stratification, prognosis and therapy response [8,9].

In recent years, studies addressing lncRNA in cancer have been gradually increasing, as they can play a very important role in several cellular processes, such as control of gene expression, through genetic and epigenetic mechanisms, chromatin remodeling, post-transcriptional regulation, and translational control [10,11]. A growing number of reports have documented that lncRNAs are abnormally expressed in several types of cancer, including breast cancer.

Several studies have identified dysregulated lncRNA expression in BRCA acting as an oncogenic (H19, UCA1, HOTAIR) [12,13,14], or exhibiting tumor suppressor functions (GAS5, BC040587, FGF14-AS2) [15,16,17]. Clearly, molecular profiling is becoming a vital tool for identifying predictive and prognostic markers for translational studies and personalized treatments. However, these treatments cause adverse effects, generate resistance, and often limit therapeutic success in patients with breast cancer [18]. An analysis using online tools showed the association of lncRNAs and miRNAs in resistance to tamoxifen (lncRNAs MALAT1 and CCAT2; miR-221, miR-222, miR-26a, miR29a, miR-29b) and trastuzumab (lncRNA GAS5, miR-16 and miR-155) [19]. On the other hand, functional studies using positive ER breast cancer cells have shown that the H19 lncRNA can confer paclitaxel chemo-resistance by silencing the BIK gene [20]. However, to date, there is no consensus on the identification of which lncRNAs expression patterns in primary BRCA are the best signature for predicting prognostic and therapeutic responses.

The identification of lncRNAs as new biomarkers and therapeutic targets for breast cancer is promising. Previously, using cDNA microarrays, we evaluated the expression profiles of MCF7 breast cancer cells expressing different levels of PAR4 before and after docetaxel exposure to identify differentially expressed genes potentially involved in PAR4-mediated chemosensitivity to docetaxel [21]. In the present study, we performed a re-analysis of that gene expression profile, aiming to identify differentially expressed lncRNAs with a potential predictive value of response to taxanes treatment for breast cancer patients. Using in silico data mining in publicly available databases such as TCGA, ROC Plotter, and KM Plotter platform, we have identified four lncRNAs as potential new biomarkers candidates for prognosis and predictive value for BRCA patients.

## 2. Materials and Methods

The studied lncRNAs were obtained from the list of differentially expressed genes (DEG) from a previous study by our group (GSE81064) [21]. The lncRNAs were manually selected among the total of 1622 up-regulated and 1308 down-regulated genes, excluding all coding RNAs and pseudogenes from the list. Initially, all the 157 differentially expressed lncRNAs (DELncRNAs; 90 up- and 67 down-regulated) were screened for differences in expression profile between normal and tumor tissues and among the different intrinsic breast cancer subtypes by using the UALCAN online tool (http://ualcan.path.uab.edu/; 22 March 2021), containing the Cancer Genome Atlas (TCGA) expression data [22]. In addition, those lncRNA were tested for impact on 120 months prognosis in terms of overall (OS) and recurrence-free survival (RFS) applying the Kaplan–Meier plotter (www.kmplot.com; 22 March 2021) [23]. Patients were stratified according to the best cut-off value for each lncRNA with the validated Jet-set probes.

**Figure 1:**
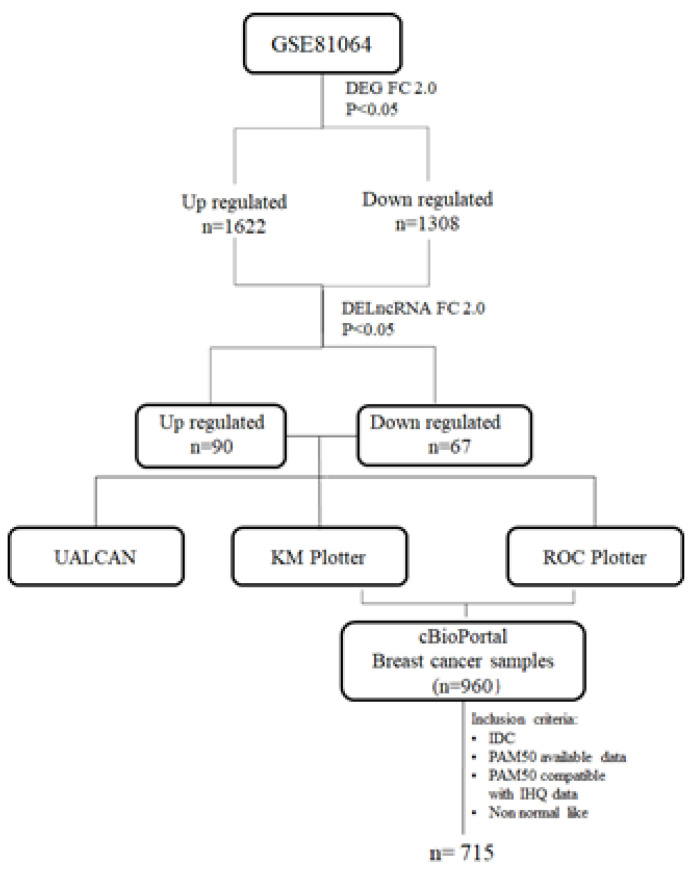
Study workflow. lncRNAs were selected among the DEGs of previous work by our group (GSE81064) using Fold change 2.0 (FC 2.0). The up- and down-regulated lncRNAs were initially screened for differences in expression, impact on prognosis, and response to taxane treatment, using the free online tools UALCAN, KMplotter, and ROC plotter, respectively. Then, the TCGA RNA expression data of the selected lncRNAs were downloaded from the cBioPortal and analyzed for the clinicopathological association.

The relation of lncRNAs expression in predicting response to taxanes was also applied for lncRNA screening by the Roc plotter tool (http://www.rocplot.org/, 22 March 2021) [24]. Top up- and down-regulated selected genes with logFC > |2| were used as input on NetworkAnalyst software [25], using second-order Network, Degree Filter > 1, and Sugiyama visualization (Figure 1).

After lncRNA selection, the TCGA expression data were downloaded from the cBioPortal for Cancer Genomics (https://www.cbioportal.org/; 22 March 2021) [26,27] and aligned with the clinicopathological data and PAM50 RNA-seq based on patients’ barcodes obtained from the Xena portal (http://xena.ucsc.edu; 22 March 2021) [28] totalizing 960 cases. For the present study, the inclusion criteria were invasive ductal carcinoma (IDC) patients and PAM50 available classification. The analyses excluded those cases with incompatibility between PAM50 classification and immunohistochemical for estrogen and progesterone receptor and, normal-like molecular subtype. In the end, a cohort of 715 patients was tested for association with clinicopathological data.

For the association between lncRNA expression and clinicopathological features, patients were categorized as low or high expression according to median lncRNA expression. The data analyses were performed by using Pearson’s chi-square test or Fisher’s exact test, as appropriate, and differences were considered statistically significant for *p*-values < 0.05 using the IBM SPSS Statistics (version 25.0; SPSS Inc., Chicago, IL, USA).

## 3. Results

### 3.1. Abnormal Expression Patterns of Four lncRNAs in BRCA Subtypes

In the present study, to identify breast cancer associated lncRNAs, we performed a re-analysis of the GSE81064 dataset obtained in a previous study from our group that aimed to evaluate the effects of PAR4 (prostate apoptosis response 4) on the expression profile of the MCF7 breast cancer cells before and after treatment with docetaxel [21]. The results revealed 157 differentially expressed lncRNAs (DELncRNAs; 90 up- and 67 down-regulated) among a total of 2930 DEGs, of which 1622 were upregulated and 1308 were downregulated. We performed in silico data mining using available public databases to investigate the potential value of the DELncRNAs as candidate biomarkers for BRCA. First, all DELncRNAs identified were evaluated using KM Plotter [23] and ROC Plotter [24] (Figure 1).

Thirteen of the 33 lncRNAs that have validated Jet-set probes in both KM Plotter and ROC Plotter were selected to construct a lncRNA network, and 4 of them (MIAT, KCNQ1OT1, LOC100270804, and FLJ10038) were highlighted as good candidate biomarkers for BRCA.

**Figure 2:**
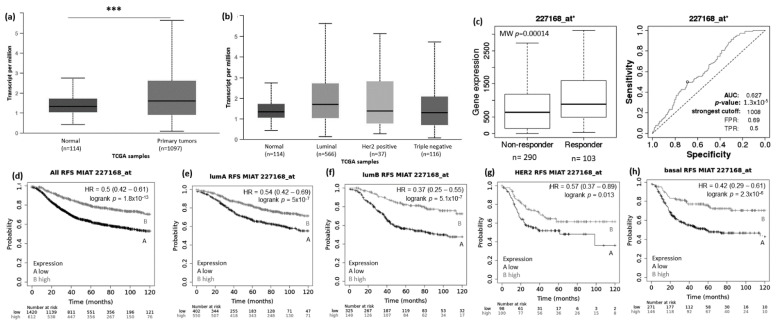
MIAT lncRNA expression in breast cancer patients and association with taxane treatment response and prognosis Expression of MIAT lncRNA in breast cancer compared to normal breast tissue (**a**) and among different breast intrinsic subtypes using the UALCAN database containing TCGA data (**b**). (**c**) MIAT lncRNA expression in groups of responder and non-responder breast cancer patients treated with taxane using the online platform ROCplot. (**d**–**h**) Kaplan–Meier curves for relapse-free survival of breast cancer patients for all subtypes (**d**) or for each intrinsic subtype as luminal A (**e**), luminal B (**f**), HER2 (**g**), and basal (**h**) grouped as high or low expression of MIAT according to the best cut-off value using the JetSet best probe set at the KMplotter online tool. *** *p* < 0.001; MW—Mann–Whitney test.

**Figure 3:**
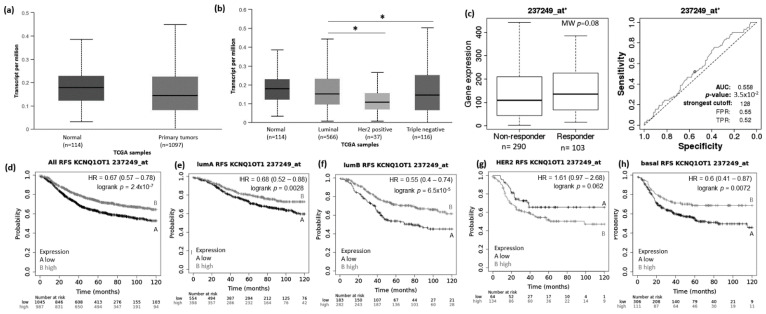
KCNQ1OT1 lncRNA expression in breast cancer patients and association with taxane treatment response and prognosis. Expression of KCNQ1OT1 lncRNA in breast cancer compared to normal breast tissue (**a**) and among different breast intrinsic subtypes using the UALCAN database containing TCGA data (**b**). (**c**) KCNQ1OT1 lncRNA expression in groups of responder and non-responder breast cancer patients treated with taxane using the online platform ROCplot. (**d**–**h**) Kaplan–Meier curves for relapse-free survival of breast cancer patients for all subtypes (**d**) or for each intrinsic subtype as luminal A (**e**), luminal B (**f**), HER2 (**g**), and basal (**h**) grouped as high or low expression of KCNQ1OT1 according to the best cut-off value using the JetSet best probe set at the KMplotter online tool. * *p* < 0.05,; MW—Mann–Whitney test.

**Figure 4:**
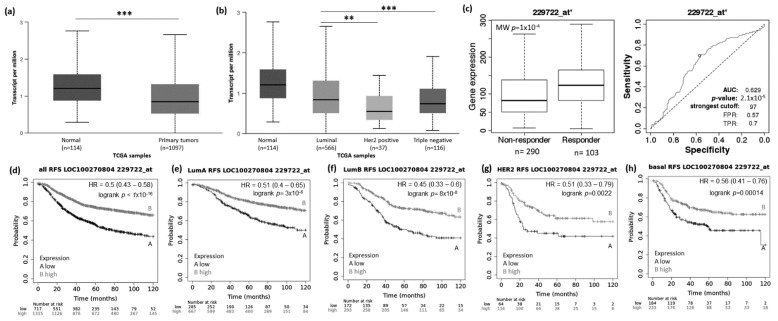
LOC100270804 lncRNA expression in breast cancer patients and association with taxane treatment response and prognosis. Expression of LOC100270804 lncRNA in breast cancer comparing to normal breast tissue (**a**) and among different breast intrinsic subtypes using the UALCAN database containing TCGA data (**b**). (**c**) LOC100270804 lncRNA expression in groups of responder and non-responder breast cancer patients treated with taxane using the online platform ROCplot. (**d**–**h**) Kaplan–Meier curves for relapse-free survival of breast cancer patients for all subtypes (**d**) or for each intrinsic subtype as luminal A (**e**), luminal B (**f**), HER2 (**g**) and basal (**h**) grouped as high or low expression of LOC100270804 according to the best cut-off value using the JetSet best probe set at the KMplotter online tool. ** *p* < 0.01, *** *p* < 0.001; MW—Mann–Whitney test.

**Figure 5:**
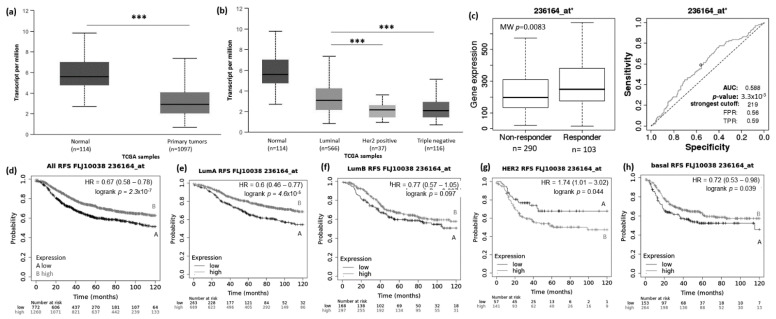
FLJ10038 lncRNA expression in breast cancer patients and association with taxane treatment response and prognosis. Expression of FLJ10038 lncRNA in breast cancer compared to normal breast tissue (**a**) and among different breast intrinsic subtypes using the UALCAN database containing TCGA data (**b**). (**c**) FLJ10038 lncRNA expression in groups of responders and non-responder breast cancer patients treated with taxane using the online platform ROCplot. (**d**–**h**) Kaplan–Meier curves for relapse-free survival of breast cancer patients for all subtypes (**d**) or each intrinsic subtype as luminal A (**e**), luminal B (**f**), HER2 (**g**), and basal (**h**) grouped as high or low expression of FLJ10038 according to the best cut-off value using the JetSet best probe set at the KMplot online tool. *** *p* < 0.001,; MW—Mann–Whitney test.

Using the UALCAN website (http://ualcan.path.uab.edu/analysis.html; 22 March 2021), we evaluated the expression pattern of MIAT, KCNQ1OT1, LOC100270804, and FLJ10038 in normal vs. BRCA tissue and among the different BRCA subtypes (luminal, HER2+, and TNBC). Results showed that the MIAT levels were higher in BRCA tissue compared to normal samples (Figure 2a), and the levels of KCNQ1OT1 (Figure 3a), LOC100270804 (Figure 4a), and FLJ10038 (Figure 5a) were lower in BRCA tissues compared to normal samples. MIAT levels showed a trend to be higher in the luminal breast cancer subtype, and KCNQ1OT1 (luminal vs. HER2+, *p* = 2.41 × 10^−2^; luminal vs. TNBC, *p* = 0.05), LOC100270804 (luminal vs. HER2+, *p* = 2.79 × 10^−2^; luminal vs. TNBC, *p* = 5.16 × 10^−3^), and FLJ10038 (luminal vs. HER2+, *p* = 2.17 × 10^−7^; luminal vs. TNBC, *p* = 4.47 × 10^−12^) median levels were found to be significantly higher in the luminal subtype.

### 3.2. lncRNAs Expression Patterns Have Impact on Patients Survival

The prognostic value of the selected DELncRNAs was evaluated using the KM Plotter platform. Lower MIAT expression conferred highly significant worse relapse-free survival (RFS) for BRCA patients, including all subtypes and also each intrinsic molecular subtype (Figure 2d–h). The impact of low MIAT expression on prognosis was also observed regarding overall survival (OS) of all breast cancer patients and the different intrinsic molecular subtypes, except for HER2+ patients (Supplementary Figure S1). Low KCNQ1OT1 expression showed RFS predictive value for all BRCA patients with tumors of the different molecular subtypes except for patients with HER2+ tumors. Conversely, high KCNQ1OT1 expression showed a trend associated with short RFS (*p* = 0.062) and significantly associated with short OS (*p* = 0.0061) in BRCA patients with tumors of the HER2+ subtype (Figure 3d–h and Supplementary Figure S2).

The low expression of LOC100270804 was significantly associated with low rates of RFS for all groups tested (Figure 4d–h, *p* < 0.01). However, for OS, the differences between the survival curves did not reach significance only for patients with tumors of the luminal B subtype (Supplementary Figure S3, *p* < 0.05).

Low expression of the lncRNA FLJ10038 was found to be associated with short rates of RFS for all BRCA patients (*p* =2.3 × 10^−7^) and for those with tumors of the luminal A (*p* = 4.6 × 10^−5^) and basal-like subtypes (*p* = 0.039). For the HER2+ subtype, the higher expression of FLJ10038 was associated with a poor prognosis (*p* = 0.044) (Figure 5e–h). For OS, the low FLJ10038 expression negatively impacts all BRCA and luminal A patients’ prognosis (Supplementary Figure S4A, *p* = 0.041 and Supplementary Figure S4B, *p* = 0.043, respectively). High expression of this lncRNA was associated with poor outcome for BRCA patients with tumors of the HER2+ and basal-like subtypes (Supplementary Figure S4D, *p* = 0.0097, and Supplementary Figure S4E, *p* = 0.034, respectively).

### 3.3. lncRNAs Expression Patterns Modulate BRCA Response to Taxane Treatment

To access the possible association between those lncRNA expressions and the response to taxane treatment, we explored in silico data on the ROC plotter platform. The diminished expression of MIAT (AUC:0.627, *p* = 1.3 × 10^−5^), LOC100270804 (AUC:0.629, *p* = 2.1 × 10^−5^), and FLJ10038 (AUC:0.588, *p* = 3.3 × 10^−3^) lncRNAs was associated with absence of complete pathological response to taxane (Figure 2c, Figure 3c, Figure 4c and Figure 5c).

### 3.4. Specific lncRNAs Expression Pattern Is Associated with Negative ER and PR BRCA Tumors

We also tested if there would be an association among those lncRNA expressions and clinicopathological features. As shown in Table 1, FLJ10038 expression displayed a strong association with ER and PR status (*p* < 0.0001). ER (OR: 0.424, CI: 0.286–0.628) and PR (OR: 0.526, CI: 0.372–0.743) negative tumors presented a lower risk of expressing high levels of FLJ10038. No other associations were found.

### 3.5. Selected lncRNAs Expression TF-Interactions Network

**Figure 6:**
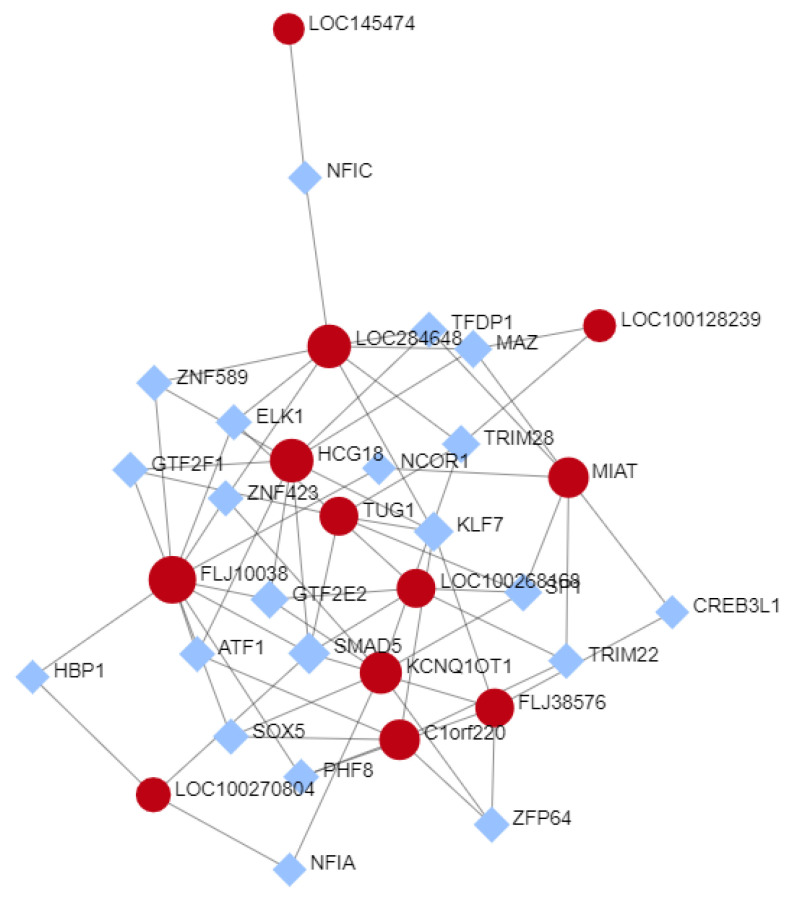
Networks of TF-lncRNAs interaction created by the NetworkAnalyst website. The lncRNA are represented as red circles, and coding mRNAs, as blue diamonds.) The nodes represent functions and edges are determined by the overlap ratio between genes associated with the two functions.

To screen the potential functional mechanism of the selected lncRNAs, a LncRNA-TF network was constructed using the NetworkAnalyst 3.0 website (https://www.networkanalyst.ca/; 22 March 2021 [25]), which utilizes KEGG pathways in its analysis. TF-gene interaction was analyzed using the ENCODE database [29]. As shown in Figure 6, several interesting TF-interactions were found. Three of the 4 lncRNAs selected as good biomarker candidates, MIAT, KCNQ1OT1, and FLJ10038, show interaction with transcription factors on the ER signaling. MIAT shows interaction with SP1 and NCOR1. KCNQ1OT1 showed interactions with SP1 and ZNF423. The FLJ10038 shows interaction with NCOR1 and ELK1. Kyoto Encyclopedia of Genes and Genomes (KEGG) pathway analysis downloaded from the NetworkAnalyst included 32 pathways significantly enriched (*p* ≤ 0.05), the top 3 significant pathways being transcriptional misregulation in cancer, TGF-β signaling pathway, and Huntington’s disease.

## 4. Discussion

Breast cancer (BRCA) remains the most prevalent neoplasia and the leading cause of cancer deaths in women worldwide [30]. Despite the progress on BRCA molecular classification, the major challenge in breast cancer care is still the identification of reliable biomarkers to determine early diagnosis, prognosis, and therapeutic response. A growing number of reports demonstrate that lncRNAs play important roles in breast cancer physiopathology and progression of breast cancer, pointing out their potential as novel molecular biomarkers for BRCA [31,32]). The present study re-evaluated the microarray dataset GSE81064 to identify lncRNA possibly associated with BRCA progression and taxanes response. We found 157 differently expressed lncRNAs (90 up-regulated and 67 down-regulated, that were manually annotated. Furthermore, after conducting data mining in silico using public databases, the results of our study pointed to 4 lncRNAs, MIAT, KCNQ1OT1, LOC100270804, and FLJ10038, with good potential as candidate biomarkers for breast cancer.

The prognostic value of the selected lncRNAs was explored using the KM Plotter platform. We found low expression of the lncRNA MIAT was associated with poor prognosis in all breast cancer subtypes. In addition, we found MIAT low expression associated with patients that do not respond to taxanes treatment. The lncRNA MIAT (myocardial infarction associated transcript) was first described as associated with susceptibility to myocardial infarction [33]. We found few reports in the literature addressing the role of MIAT in breast cancer. Up-regulation of MIAT has been found in breast cancer cell lines and breast tumor samples compared to the normal breast tissue [34,35,36]. Li et al. (2020) evaluated the expression of MIAT breast cancer cases and found that its expression was associated with poor prognosis [37]. They also provided experimental evidence for the mechanism by which MIAT influences the progression of BRCA, showing that MIAT overexpressed in breast cancer cells promotes invasion, migration, and DLG3 promoter methylation. Knockdown of MIAT inhibited breast cancer cell proliferation, migration, invasion, and EMT and increased the rate of apoptosis in breast cancer cells [34,35,36]. Furthermore, down regulation of MIAT decreased tumor growth and delayed tumor formation in vivo, suggesting that MIAT might promote breast cancer malignant progression [34,37]. Contrary to our results, taken together, these findings suggest that MIAT functions as an oncogene and highlight that further clinical and experimental studies are required to define better the role and potential prognostic value of MIAT expression in BRCA. MIAT expression was found up-regulated in ER-positive breast cancer cell line MCF-7 as compared to ER-negative breast cancer cell line MDA-MB-231 and was induced by estrogen in a dose- and time-dependent fashion via ER in the MCF-7 cells [38]. Here, we found that MIAT expression was higher in breast tumors of the luminal subtype compared to the other subtypes. We also found that MIAT low expression confers poor prognosis for BRCA patients of the luminal subtype (luminal A and luminal B). Interestingly, we generated a TF-interaction network (Network Analyst website) and found that MIAT interacts with NCOR1 and SP1, which are important components of the machinery involved in ER mechanism of action [39,40,41]. (These findings on the connection of MIAT to the mechanism of ER action could suggest that its expression might impact endocrine therapy resistance and need to be further explored.

lncRNA KCNQ10T1 is located in the chromosomal region 11p15.5, which is rich in epigenetically regulated genes and expressed preferentially from a parental chromosome. The main function of the lncRNA KCNQ10T1 is to regulate the expression of the KCNQ1 gene (potassium voltage-gated channel subfamily Q member 1) through interaction with chromatin, in addition to the transcriptional regulation of 8 target maternally expressed genes [42]. This lncRNA has been described as an oncogene in different types of tumors. The increase in its expression has been correlated with a worse prognosis in non-small cell lung tumors (NSCLC) [43]. In addition, a direct relationship between the increase in KCNQ10T1 and the proliferation and migration of bladder carcinoma cells was observed through the modulation of mir-145-5p/PCBP2 [44]. In SKBR3 breast cancer lineage, silencing of KCNQ10T1 showed an association with reduced proliferation, migration, invasion and epithelial-mesenchymal transition, and induction of apoptosis. In breast tumors, Feng et al. (2018) showed that the expression of KCNQ1OT1 was much higher in cancerous breast tissues (n = 18) than in normal adjacent breast tissues and that the increase in KCNQ10T1 showed an association with the advanced tumor stage (III/IV) [45]. Our analysis of the TCGA data showed a reduction in lncRNA KCNQ10T1 in breast tumors compared to normal tissue. We have to emphasize that TCGA has a large cohort of breast tumors; however, the normal tissues were not matched by tumor tissue of the same patient. However, when we stratified the analyses, considering breast tumor subtypes, lncRNA KCNQ10T1 was more expressed in luminal and basal subtype tumors. The relationship with survival showed that patients with tumors of luminal subtype A, luminal B, and basal with low expression of KCNQ10T1 have lower RFS, while high expression of KCNQ10T1 in patients with tumors of subtype HER2+ was associated with short overall survival. This increase in KCNQ10T1 expression has also been described in triple-negative tumors in relation to adjacent normal tissue. In addition, the increase in KCNQ10T1 was associated with a decrease in PTEN in strains leading to the development of TNBC [46]. Rodriguez et al. (2011) showed that the treatment of MCF7 cells with E2 induced the expression of KCNQ10T1, suggesting an expression control mediated by the estrogen signaling pathway [47]. In the TF-interaction network, we found that KCNQ1OT1 shows interactions with SP1 and ZNF423. Both ER α and ER β are able to interact with the Sp1 protein to transactivate target genes through a pathway for hormone activation of genes in which the receptor does not directly bind DNA but plays an important role in transcriptional activation of multiple growth regulatory genes in breast cancer cells [39]. ZNF423 is a zinc finger transcription factor related to the E2-dependent induction of BRCA1 [48] and an intronic single nucleotide polymorphism (SNP) in ZNF423, rs9940645, determines tamoxifen response [44,49]. These findings provide new insights into the connection between KCNQ1OT1 and the estrogen signaling pathways that need further investigation.

We also identified two novel breast cancer-associated lncRNAs, whose expression pattern showed the potential to be promising candidate biomarkers for BRCA. The lncRNAs FLJ10038 (GABPB1-IT1) and LOC100270804 (LINC00653) were significantly down-regulated in breast tumor samples as compared to normal samples in the TGCA dataset. Low levels of LOC100270804 were associated with short RFS for breast cancer patients with all intrinsic subtypes. Low expression of the FLJ10038 confers short RFS rates, especially for breast cancer patients with tumors of the luminal A subtype. For FLJ10038 and LOC100270804, high expression showed potential predictive value for taxane treatment response (ROC Plotter dataset). Xie J et al. (2019) showed that GABPB1-IT1 expression was significantly downregulated in lung cancer [50]. Its expression was lower in high-grade NSCLC samples than low-grade NSCLC samples. They also found that low GABPB1-IT1 expression levels were associated with poor survival of patients with NSCLC. So far, we have not identified any other reports in the literature assessing the functional role or predictive value of these two lncRNAs in breast cancer or other cancers. The current study is the first to provide insights into FLJ10038 and LOC100270804 as potential candidate biomarkers for BRCA. However, more clinical and experimental studies are needed to evaluate the functional role of these lncRNAs and their feasibility as a biomarker for breast cancer patients.

## 5. Conclusions

In conclusion, based on the re-analysis of previous microarray datasets from our group and in silico analysis, our findings provide new insights on the involvement of lncRNAs MIAT, KCNQ1OT1, LOC100270804, and FLJ10038 in the pathogenesis of BRCA. Further clinical and experimental studies are required to delineate the functional role and prognostic and predictive value of these lncRNAs in breast cancer.

## Figures and Tables

**Table 1:** Association of lncRNA expression with clinicopathological characteristics of primary ductal infiltrative breast cancer patients (Cbio Portal—TCGA data).

Characteristic	Category	MIAT			KCNQ1OT1			FLJ10038			LOC100270804		
		Low n (%)	High n (%)	*p*-Value	Low n (%)	High n (%)	*p*-Value	Low n (%)	High n (%)	*p*-value	Low n (%)	High n (%)	*p*-Value
Age (years)	<50 y	42 (6.7)	144 (23.1)	0.697	94 (15.1)	92 (14.8)	0.644	93 (14.9)	93 (14.9)	0.814	31 (5.0)	155 (24.9)	0.534
	≥50 y	105 (19.9)	332 (53.3)		212 (34.0)	225 (36.1)		214 (34.3)	223 (35.8)		82 (13.2)	355 (57.0)	
Tumor Size	Early	125 (20.1)	412 (66.2)	0.599	270 (43.4)	267 (42.9)	0.174	265 (42.6)	272 (43.7)	0.849	96 (15.4)	441 (70.9)	0.637
	late	22 (3.5)	63 (10.1)		36 (5.8)	49 (7.9)		41 (6.6)	44 (7.1)		17 (2.7)	68 (10.9)	
Nodal status	pN	67 (11.0)	216 (35.4)	0.900	136 (22.3)	147 (24.1)	0.606	143 (23.4)	140 (23.0)	0.639	53 (8.7)	230 (37.7)	0.752
	pN+	76 (12.5)	251 (41.1)		164 (26.9)	163 (26.7)		159 (26.1)	168 (27.5)		58 (9.5)	269 (44.1)	
Presence of Metastasis	pM	133 (23.3)	424 (74.4)	1.000	267 (46.8)	290 (50.9)	0.076	278 (48.8)	279 (48.9)	0.779	101 (17.7)	456 (80.0)	0.332
	pM+	3 (0.5)	10 (1.8)		3 (0.5)	10 (1.8)		7 (1.2)	6 (1.1)		1 (0.2)	12 (2.1)	
Tumor Stage	Early	108 (17.8)	361 (59.6)	0.541	224 (37.5)	245 (40.4)	0.323	225 (37.1)	244 (40.3)	0.274	84 (13.9)	385 (63.5)	0.632
	late	35 (5.8)	102 (16.8)		72 (11.9)	65 (10.7)		73 (12.0)	64 (10.6)		27 (4.5)	110 (18.2)	
ER status	negative	41 (6.9)	99 (16.7)	0.093	67 (11.3)	73 (12.3)	0.691	91 (15.4)	49 (8.3)	<0.0001	32 (5.4)	108 (18.2)	0.154
	positive	101 (17.1)	351 (59.3)		225 (38.0)	227 (38.3)		199 (33.6)	253 (42.7)		79 (13.3)	373 (63.0)	
PR status	negative	56 (9.5)	144 (24.4)	0.081	90 (15.3)	110 (18.6)	0.118	119 (20.2)	81 (13.7)	<0.0001	43 (7.3)	157 (26.6)	0.232
	positive	84 (14.2)	306 (51.9)		202 (34.2)	188 (31.9)		170 (28.8)	220 (37.3)		68 (11.5)	322 (54.6)	
Menopausal Status	pre	28 (5.3)	105 (19.7)	0.486	67 (12.6)	66 (12.4)	0.708	62 (11.6)	71 (13.3)	0.706	23 (4.3)	110 (20.6)	0.905
	post	96 (18.0)	304 (57.0)		194 (36.4)	206 (28.6)		194 (36.4)	206 (38.6)		71 (13.3)	329 (61.7)	

Chi-square or Fisher’s exact test was used.

## Data Availability

All data that support the findings of this study are available from the corresponding author, upon request.

## Supplementary Materials

The following are available online at https://www.mdpi.com/article/10.3390/genes12070996/s1, Supplementary Figure S1. Kaplan-Meier curves for long-term overall (OS) of breast cancer patients stratified according to MIAT lncRNA expression; Supplementary Figure S2. Kaplan-Meier curves for long-term overall (OS) of breast cancer patients stratified according to KCNQ1OT1 lncRNA expression; Supplementary Figure S3. Kaplan-Meier curves for long-term overall (OS) of breast cancer patients stratified according to LOC100270804 lncRNA expression; Supplementary Figure S4. Kaplan-Meier curves for long-term overall (OS) of breast cancer patients stratified according to FLJ10038 lncRNA expression.

## References

[1] Siegel R.L., Miller K.D., Fuchs H.E., Jemal A. (2021). Cancer Statistics, 2021. CA Cancer J. Clin..

[2] Allemani C., Matsuda T., Di Carlo V., Harewood R., Matz M., Nikšić M., Bonaventure A., Valkov M., Johnson C.J., Estève J. (2018). Global surveillance of trends in cancer survival 2000-14 (CONCORD-3): Analysis of individual records for 37 513 025 patients diagnosed with one of 18 cancers from 322 population-based registries in 71 countries. Lancet.

[3] Perou C.M., Sørlie T., Eisen M.B., Van De Rijn M., Jeffrey S.S., Rees C.A., Pollack J.R., Ross D.T., Johnsen H., Akslen L.A. (2000). Molecular portraits of human breast tumours. Nature.

[4] Sørlie T., Perou C.M., Tibshirani R., Aas T., Geisler S., Johnsen H., Hastie T., Eisen M.B., van de Rijn M., Jeffrey S.S. (2001). Gene expression patterns of breast carcinomas distinguish tumor subclasses with clinical implications. Proc. Natl. Acad. Sci. USA.

[5] Cech T.R., Steitz J.A. (2014). The Noncoding RNA Revolution—Trashing Old Rules to Forge New Ones. Cell.

[6] Romano G., Veneziano D., Acunzo M., Croce C.M. (2017). Small non-coding RNA and cancer. Carcinogenesis.

[7] Kopp F., Mendell J.T. (2018). Functional Classification and Experimental Dissection of Long Noncoding RNAs. Cell.

[8] Bhan A., Soleimani M., Mandal S.S. (2017). Long Noncoding RNA and Cancer: A New Paradigm. Cancer Res..

[9] Chi Y., Wang D., Wang J., Yu W., Yang J. (2019). Long Non-Coding RNA in the Pathogenesis of Cancers. Cells.

[10] Perry R.B., Ulitsky I. (2016). The functions of long noncoding RNAs in development and stem cells. Development.

[11] Wang J., Ye C., Xiong H., Shen Y., Lu Y., Zhou J., Wang L. (2017). Dysregulation of long non-coding RNA in breast cancer: An overview of mechanism and clinical implication. Oncotarget.

[12] Barsyte-Lovejoy D., Lau S.K., Boutros P.C., Khosravi F., Jurisica I., Andrulis I.L., Tsao M.S., Penn L.Z. (2006). The c-Myc oncogene directly induces the H19 noncoding RNA by allele-specific binding to potentiate tumorigenesis. Cancer Res..

[13] Huang J., Zhou N., Watabe K., Lu Z., Wu F., Xu M., Mo Y.Y. (2014). Long non-coding RNA UCA1 promotes breast tumor growth by suppression of p27 (Kip1). Cell Death Dis..

[14] Hajjari M., Salavaty A. (2015). HOTAIR: An oncogenic long non-coding RNA in different cancers. Cancer Biol. Med..

[15] Chi Y., Huang S., Yuan L., Liu M., Huang N., Zhou S., Zhou B., Wu J. (2014). Role of BC040587 as a predictor of poor outcome in breast cancer. Cancer Cell Int..

[16] Pickard M.R., Williams G.T. (2016). The hormone response element mimic sequence of GAS5 lncRNA is sufficient to induce apoptosis in breast cancer cells. Oncotarget.

[17] Xu S., Wang P., You Z., Meng H., Mu G., Bai X., Zhang G., Zhang J., Pang D. (2016). The long non-coding RNA EPB41L4A-AS2 inhibits tumor proliferation and is associated with favorable prognoses in breast cancer and other solid tumors. Oncotarget.

[18] Milani A., Geuna E., Mittica G., Valabrega G. (2014). Overcoming endocrine resistance in metastatic breast cancer: Current evidence and future directions. World J. Clin. Oncol..

[19] Corrà F., Agnoletto C., Minotti L., Baldassari F., Volinia S. (2018). The Network of Non-coding RNAs in Cancer Drug Resistance. Front. Oncol..

[20] Si X., Zang R., Zhang E., Liu Y., Shi X., Shao L., Li A., Yang N., Han X., Pan B. (2016). LncRNA H19 confers chemoresistance in ERα-positive breast cancer through epigenetic silencing of the pro-apoptotic gene BIK. Oncotarget.

[21] de Bessa Garcia S.A., Pavanelli A.C., Cruz E Melo N., Nagai M.A. (2017). Prostate apoptosis response 4 (PAR4) expression modulates WNT signaling pathways in MCF7 breast cancer cells: A possible mechanism underlying PAR4-mediated docetaxel chemosensitivity. Int. J. Mol. Med..

[22] Tang Z., Li C., Kang B., Gao G., Zhang Z. (2017). GEPIA: A web server for cancer and normal gene expression profiling and interactive analyses. Nucleic Acids Res..

[23] Nagy Á., Lánczky A., Menyhárt O., Győrffy B. (2018). Validation of miRNA prognostic power in hepatocellular carcinoma using expression data of independent datasets. Sci. Rep..

[24] Fekete J.T., Győrffy B. (2019). ROCplot.org: Validating predictive biomarkers of chemotherapy/hormonal therapy/anti-HER2 therapy using transcriptomic data of 3,104 breast cancer patients. Int. J. Cancer.

[25] Zhou G., Soufan O., Ewald J., Hancock R.E.W., Basu N., Xia J. (2019). NetworkAnalyst 3.0: A visual analytics platform for comprehensive gene expression profiling and meta-analysis. Nucleic Acids Res..

[26] Cerami E., Gao J., Dogrusoz U., Gross B.E., Sumer S.O., Aksoy B.A., Jacobsen A., Byrne C.J., Heuer M.L., Larsson E. (2012). The cBio cancer genomics portal: An open platform for exploring multidimensional cancer genomics data. Cancer Discov..

[27] Gao J., Aksoy B.A., Dogrusoz U., Dresdner G., Gross B., Sumer S.O., Sun Y., Jacobsen A., Sinha R., Larsson E. (2013). Integrative analysis of complex cancer genomics and clinical profiles using the cBioPortal. Sci. Signal..

[28] Goldman M.J., Craft B., Hastie M., Repečka K., McDade F., Kamath A., Banerjee A., Luo Y., Rogers D., Brooks A.N. (2020). Visualizing and interpreting cancer genomics data via the Xena platform. Nat. Biotechnol..

[29] Moore J.E., Purcaro M.J., Pratt H.E., Epstein C.B., Shoresh N., Adrian J., Kawli T., Davis C.A., Dobin A., Kaul R. (2020). Expanded encyclopaedias of DNA elements in the human and mouse genomes. Nature.

[30] Bray F., Ferlay J., Soerjomataram I., Siegel R.L., Torre L.A., Jemal A. (2018). Global cancer statistics 2018: GLOBOCAN estimates of incidence and mortality worldwide for 36 cancers in 185 countries. CA Cancer J Clin.

[31] Richard J.L.C., Eichhorn P.J.A. (2018). Deciphering the roles of lncRNAs in breast development and disease. Oncotarget.

[32] Zhou S., He Y., Yang S., Hu J., Zhang Q., Chen W., Xu H., Zhang H., Zhong S., Zhao J. (2018). The regulatory roles of lncRNAs in the process of breast cancer invasion and metastasis. Biosci. Rep..

[33] Ishii N., Ozaki K., Sato H., Mizuno H., Susumu S., Takahashi A., Miyamoto Y., Ikegawa S., Kamatani N., Hori M. (2006). Identification of a novel non-coding RNA, MIAT, that confers risk of myocardial infarction. J. Hum. Genet..

[34] Luan T., Zhang X., Wang S., Song Y., Zhou S., Lin J., An W., Yuan W., Yang Y., Cai H. (2017). Long non-coding RNA MIAT promotes breast cancer progression and functions as ceRNA to regulate DUSP7 expression by sponging miR-155-5p. Oncotarget.

[35] Alipoor F.J., Asadi M.H., Torkzadeh-Mahani M. (2018). MIAT lncRNA is overexpressed in breast cancer and its inhibition triggers senescence and G1 arrest in MCF7 cell line. J. Cell Biochem..

[36] Almnaseer Z.A., Mourtada-Maarabouni M. (2018). Long noncoding RNA MIAT regulates apoptosis and the apoptotic response to chemotherapeutic agents in breast cancer cell lines. Biosci. Rep..

[37] Li D., Hu X., Yu S., Deng S., Yan M., Sun F., Song J., Tang L. (2020). Silence of lncRNA MIAT-mediated inhibition of DLG3 promoter methylation suppresses breast cancer progression via the Hippo signaling pathway. Cell Signal..

[38] Li Y., Jiang B., Wu X., Huang Q., Chen W., Zhu H., Qu X., Xie L., Ma X., Huang G. (2018). Long non-coding RNA MIAT is estrogen-responsive and promotes estrogen-induced proliferation in ER-positive breast cancer cells. Biochem. Biophys. Res. Commun..

[39] Safe S. (2001). Transcriptional activation of genes by 17 β-estradiol through estrogen receptor-Sp1 interactions. Vitam. Horm..

[40] Green K.A., Carroll J.S. (2007). Oestrogen-receptor-mediated transcription and the influence of co-factors and chromatin state. Nat. Rev. Cancer.

[41] Bartella V., Rizza P., Barone I., Zito D., Giordano F., Giordano C., Catalano S., Mauro L., Sisci D., Panno M.L. (2012). Estrogen receptor β binds Sp1 and recruits a corepressor complex to the estrogen receptor α gene promoter. Breast Cancer Res. Treat..

[42] Mohammad F., Pandey R.R., Nagano T., Chakalova L., Mondal T., Fraser P., Kanduri C. (2008). Kcnq1ot1/Lit1 noncoding RNA mediates transcriptional silencing by targeting to the perinucleolar region. Mol. Cell. Biol..

[43] Dong Z., Yang P., Qiu X., Liang S., Guan B., Yang H., Li F., Sun L., Liu H., Zou G. (2019). KCNQ1OT1 facilitates progression of non-small-cell lung carcinoma via modulating miRNA-27b-3p/HSP90AA1 axis. J. Cell Physiol..

[44] Wang G., Qin S., Zayas J., Ingle J.N., Liu M., Weinshilboum R.M., Shen K., Wang L. (2019). 4-Hydroxytamoxifen enhances sensitivity of estrogen receptor α-positive breast cancer to docetaxel in an estrogen and ZNF423 SNP-dependent fashion. Breast Cancer Res. Treat..

[45] Feng W., Wang C., Liang C., Yang H., Chen D., Yu X., Zhao W., Geng D., Li S., Chen Z. (2018). The Dysregulated Expression of KCNQ1OT1 and Its Interaction with Downstream Factors miR-145/CCNE2 in Breast Cancer Cells. Cell Physiol. Biochem..

[46] Shen B., Li Y., Ye Q., Qin Y. (2020). YY1-mediated long non-coding RNA Kcnq1ot1 promotes the tumor progression by regulating PTEN via DNMT1 in triple negative breast cancer. Cancer Gene Ther..

[47] Rodriguez B.A., Weng Y.I., Liu T.M., Zuo T., Hsu P.Y., Lin C.H., Cheng A.L., Cui H., Yan P.S., Huang T.H. (2011). Estrogen-mediated epigenetic repression of the imprinted gene cyclin-dependent kinase inhibitor 1C in breast cancer cells. Carcinogenesis.

[48] Ingle J.N., Liu M., Wickerham D.L., Schaid D.J., Wang L., Mushiroda T., Kubo M., Costantino J.P., Vogel V.G., Paik S. (2013). Selective estrogen receptor modulators and pharmacogenomic variation in ZNF423 regulation of BRCA1 expression: Individualized breast cancer prevention. Cancer Discov..

[49] Bond H.M., Scicchitano S., Chiarella E., Amodio N., Lucchino V., Aloisio A., Montalcini Y., Mesuraca M., Morrone G. (2018). ZNF423: A New Player in Estrogen Receptor-Positive Breast Cancer. Front. Endocrinol..

[50] Xie J., Xie G., Chen Q., Xu Z., Bai W., Chen M. (2019). Identification of a novel lncRNA GABPB1-IT1 that is downregulated and predicts a poor prognosis in non-small cell lung cancer. Oncol. Lett..

