# Supplementary figures and images for: Abnormal Long Non-Coding RNAs Expression Patterns Have the Potential Ability for Predicting Survival and Treatment Response in Breast Cancer
Source: Genes (Basel). 2021 Jun 29;12(7):996. doi: 10.3390/genes12070996 (PMC8305383; doi:10.3390/genes12070996)

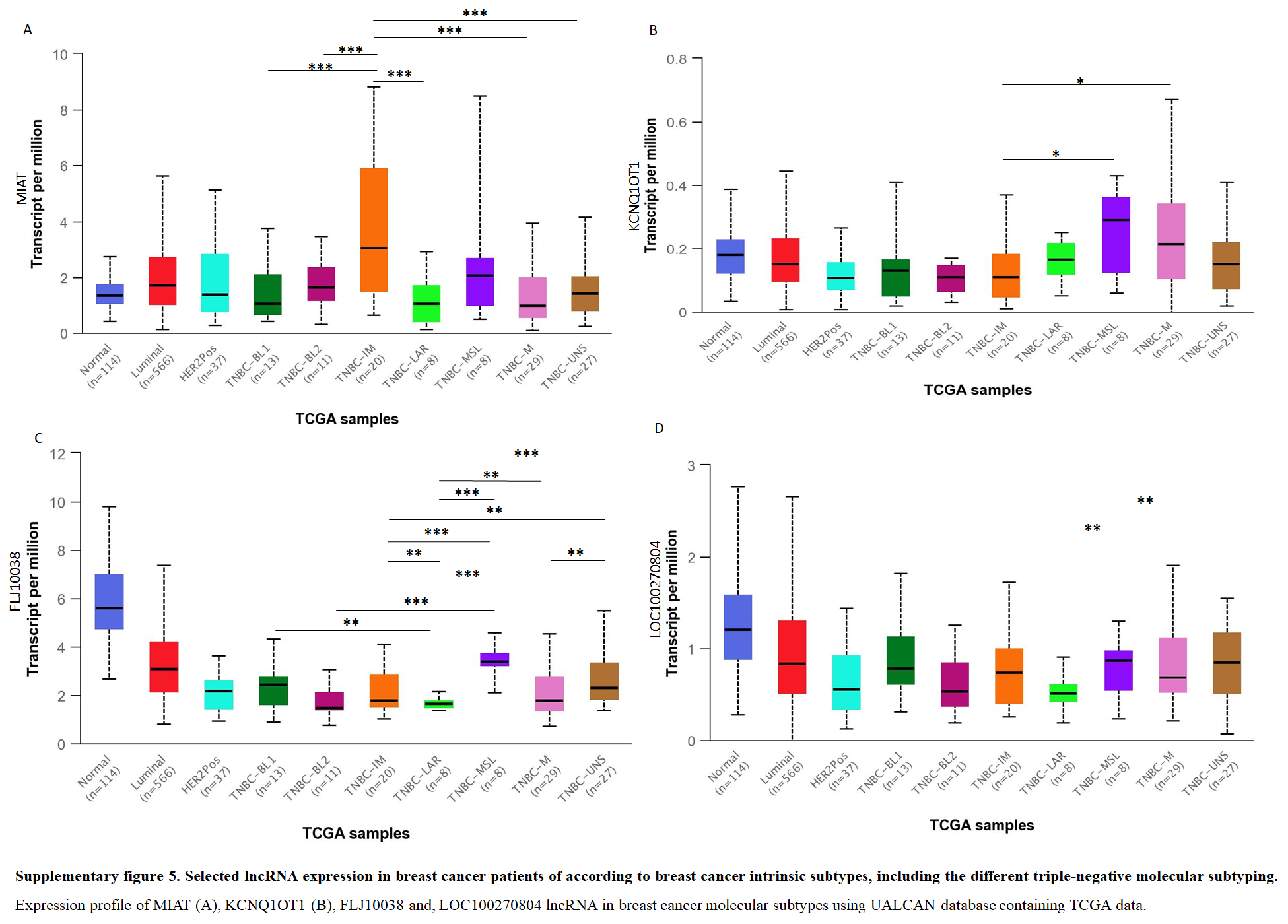

Supplement: Supplementary file 1 [file genes-12-00996-s001.zip › Supplementary Figure 5.tiff]

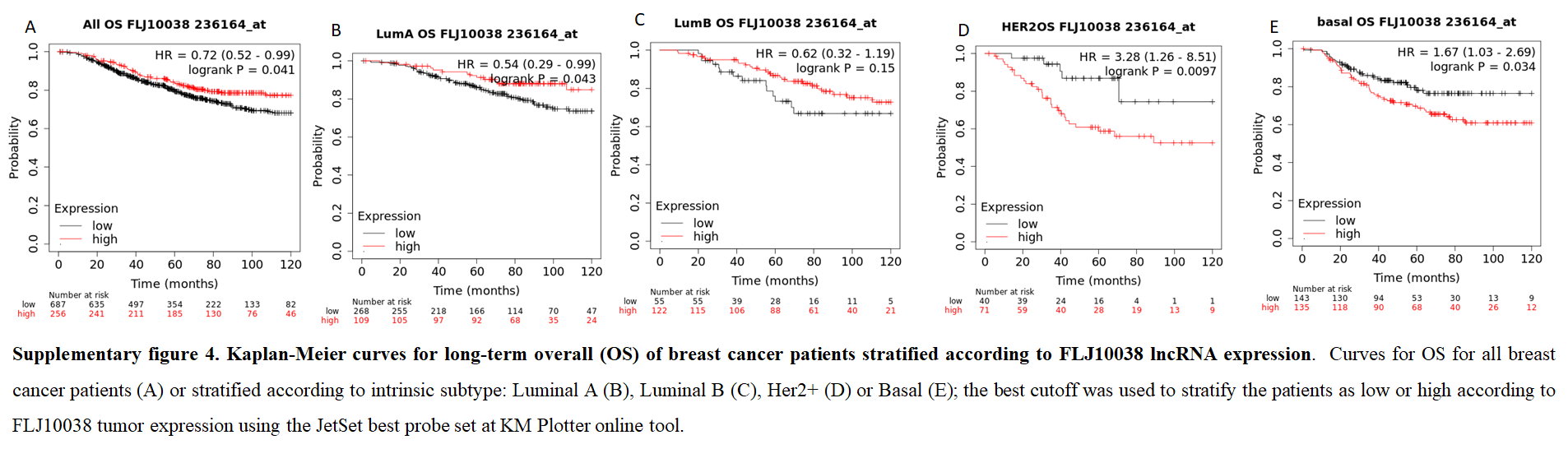

Supplement: Supplementary file 1 [file genes-12-00996-s001.zip › Supplementary Figure 4.tiff]

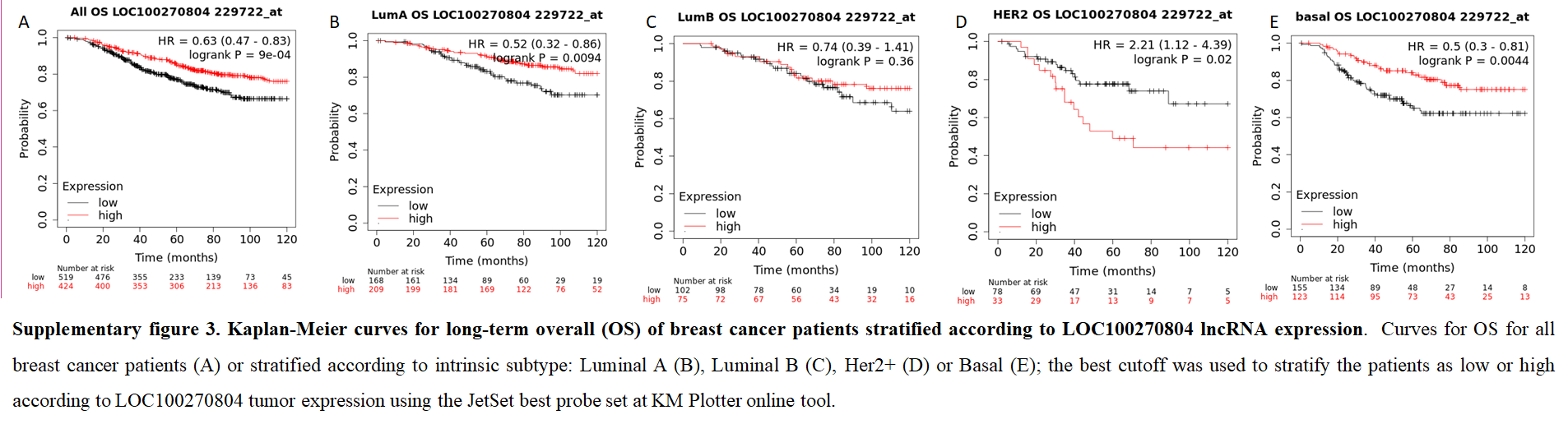

Supplement: Supplementary file 1 [file genes-12-00996-s001.zip › Supplementary Figure 3.tiff]

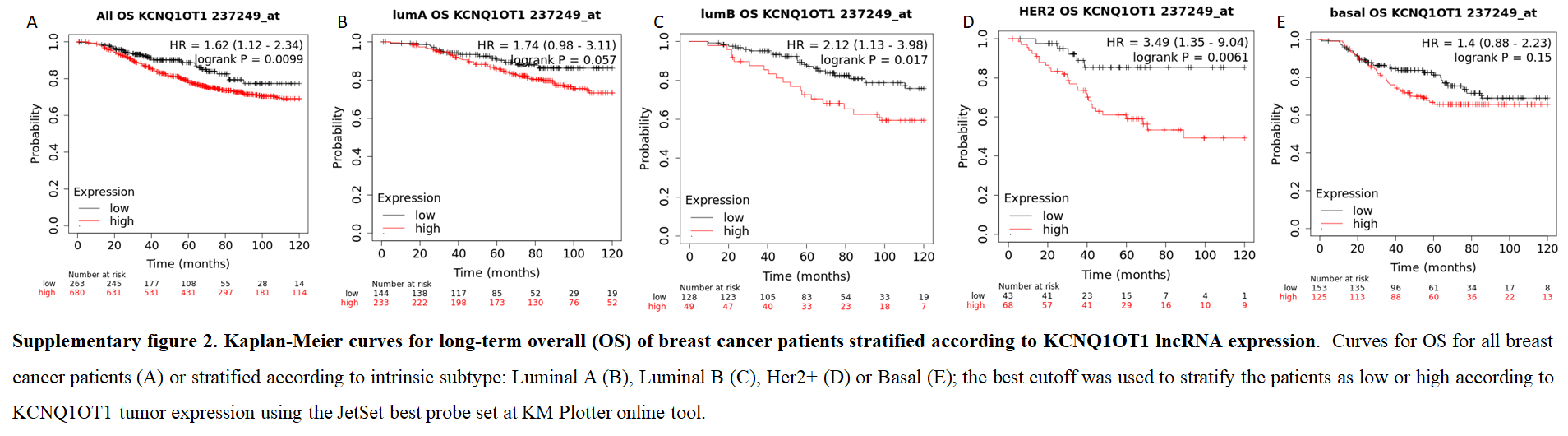

Supplement: Supplementary file 1 [file genes-12-00996-s001.zip › Supplementary Figure 2.tiff]

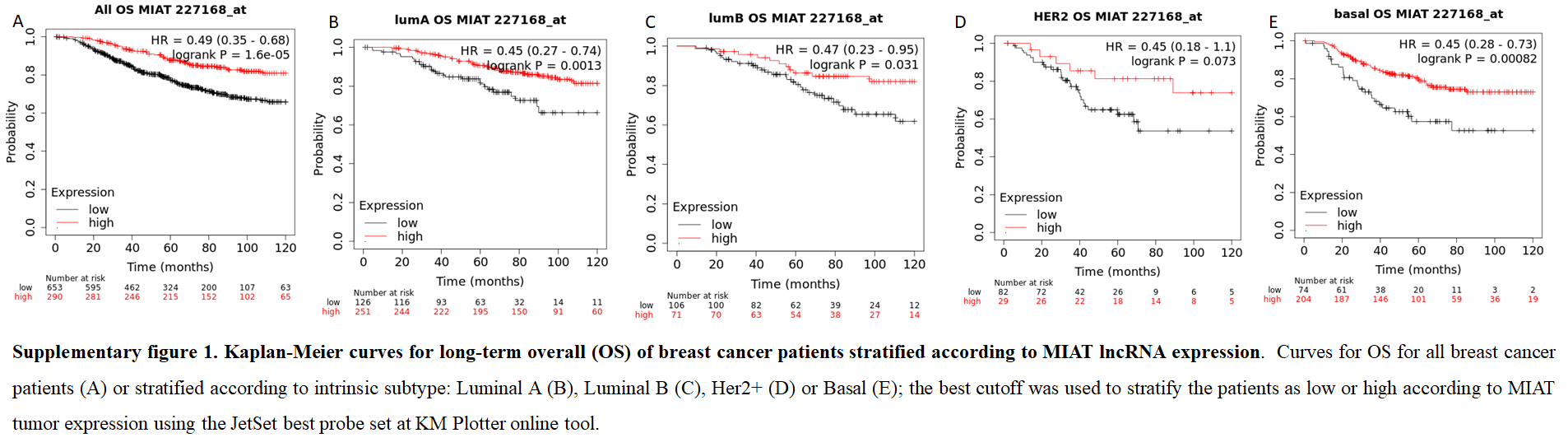

Supplement: Supplementary file 1 [file genes-12-00996-s001.zip › Supplementary Figure 1 .tiff]
